# Improving Selective Attention in Healthy Adults Through SMR Neurofeedback: Electrophysiological and Behavioral Evidence

**DOI:** 10.3390/brainsci16080843

**Published:** 2026-08-08

**Authors:** Ivana Stanković, Ljiljana Jeličić, Jelena Đorđević, Nela Ilić, Mirjana Sovilj, Slavica Maksimović, Maša Marisavljević, Miško Subotić

**Affiliations:** 1 Cognitive Neuroscience Department, Research and Development Institute “Life Activities Advancement Institute”, 11000 Belgrade, Serbia; 2 Department of Speech, Language and Hearing Sciences, Institute for Experimental Phonetics and Speech Pathology, 11000 Belgrade, Serbia; 3 Clinic for Neurology and Psychiatry for Children and Adolescents, 11000 Belgrade, Serbia; 4 Department of Psychiatry, Faculty of Medical Sciences, University of Kragujevac, 34000 Kragujevac, Serbia; 5 Faculty of Medicine, University of Belgrade, 11000 Belgrade, Serbia; 6 Clinic for Physical Medicine and Rehabilitation, 11000 Belgrade, Serbia

**Keywords:** neurofeedback, sensorimotor rhythm, selective auditory attention, event-related potentials (N100; N200; P300), cognitive enhancement

## Abstract

**Background/Objectives:** Neurofeedback (NFB) modulates electroencephalographic activity and has been proposed to enhance cognitive performance. This study investigated the effects of repeated sensorimotor rhythm (SMR; 12–15 Hz) neurofeedback training on selective auditory attention in healthy adults aged 25–40 years, utilizing both electrophysiological and behavioral assessments. **Methods:** A prospective, controlled factorial design with repeated measures was employed, including a sham NFB intervention to control for placebo effects. Electrophysiological evaluation measured the amplitudes and latencies of event-related potentials (N100, N200, and P300) during an auditory Go/No-Go task. Speech-in-noise perception, which demands a higher level of selective auditory attention, was further assessed using the QuickSIN test. **Results:** Electrophysiological results revealed that SMR training statistically significantly reduced latencies of the N200 component at the Cz and Pz electrodes, and the P300 component at the Fz, Cz, and Pz electrodes, in a dose-dependent manner. No significant amplitude effects were observed for any ERP component. Behavioral results demonstrated a linear improvement in speech perception in noise (QuickSIN test) in the experimental group, a task that indirectly engages auditory selective attention mechanisms, as a function of training sessions, with significant group differences favoring the experimental group. These effects were maintained at the one-month follow-up. **Conclusions:** These findings indicate that SMR-based NFB modulates neural activity and improves selective attention, supporting its potential as an effective intervention for improving attentional performance and auditory processing in healthy populations, with effects persisting at least one month post-training.

## 1. Introduction

Enhancing cognitive performance is of growing interest in multidisciplinary research due to its impact on individual, social, and economic functioning. Higher cognitive functions, particularly attention, underpin daily activities, academic achievement, and the effective operation of other cognitive processes. Attention allows individuals to focus on relevant external or internal information while filtering out irrelevant input, and its proper functioning is critical for effective information processing [1,2,3]. Selective attention, in particular, is required when multiple stimuli compete for processing, enabling the prioritization of relevant information while ignoring distractions. Neurophysiologically, attention is associated with increased cortical activation and modulation of event-related potentials (ERPs), such as the N100, N200, and P300 components, which reflect the brain’s processing of sensory and cognitive information [4]. Although these ERP components are well-established markers of attentional processes, the extent to which they can be modulated through non-invasive interventions such as neurofeedback and particularly sensorimotor rhythm (SMR) training has not been systematically investigated in healthy adults, especially within the 25–40 age range, a population often underrepresented in cognitive enhancement research despite being at a life stage where cognitive demands, both professional and personal, are typically high.

Extensive research has advanced our understanding of how auditory attention influences speech perception in complex acoustic environments [5,6]. The ability to direct auditory attention despite numerous distractors, known as the “cocktail party effect” [7], involves cognitive processes that allow the brain to focus on a single sound stream, often speech, while suppressing irrelevant background noise. Selective auditory attention enables the identification of a specific sound stimulus among multiple simultaneous sources and is crucial for everyday communication and understanding speech in noisy environments [5]. Importantly, individual differences in auditory selective attention exist even among young listeners with normal hearing [8,9]. Given that these attentional processes are reflected in modulations of ERPs such as the N100, N200, and P300, they present a viable target for non-invasive interventions.

Electroencephalography (EEG) enables monitoring of brain activity during cognitive tasks, providing insight into the dynamics of human brain function [10]. Furthermore, event-related potentials (ERPs), which are time-locked EEG responses to specific sensory, motor, or cognitive events, capture changes in neural activity evoked either directly by external stimuli (exogenous potentials) or by cognitive processing of these stimuli (endogenous potentials) [4,11]. Endogenous components, recorded during cognitive tasks, reflect higher-level processes such as memory, attention, and cognitive control [4,12]. Among these, the N100, N200, and P300 components are widely used as objective indices of perceptual and higher-order cognitive processes, including attention, inhibitory control, and memory updating [13]. ERP amplitude is generally interpreted as reflecting the magnitude of underlying neuronal activity, whereas latency is considered an index of processing speed [4]. In particular, the P300 component has been extensively associated with attentional resource allocation and stimulus evaluation processes, with larger amplitudes and shorter latencies typically indicating more efficient cognitive processing [14,15].

The brain is capable of functional and structural adaptation, a property known as neuroplasticity, which enables it to adjust in response to environmental demands, physiological changes, and experience [16,17]. Evidence indicates that neuroplasticity persists throughout the lifespan, motivating the development of neuromodulation techniques to enhance cognitive performance in both healthy and clinical populations. Neurofeedback (NFB) is one such method, in which participants learn to voluntarily modulate their brain activity through real-time EEG feedback [18]. Unlike other neuromodulation approaches, such as transcranial magnetic stimulation (TMS) and transcranial electrical stimulation (TES), neurofeedback (NFB) relies on active participant engagement and establishes a functional link between cortical oscillatory activity and cognitive processes, potentially supporting cognitive improvements through self-regulation. As a form of behavioral training, NFB is thought to promote functional reorganization via neuroplasticity and relies on continuous EEG monitoring and real-time feedback, enabling individuals to learn to modulate specific brain oscillations and achieve desired mental states [19]. Although research on EEG-based modulation dates back to the 1930s, systematic investigations emerged in the 1960s and 1970s, providing early evidence for the voluntary modulation of brain activity and its potential to enhance sensorimotor rhythms and alleviate symptoms in neurological disorders, with later research and reviews further examining its clinical potential [20,21,22]. However, methodological challenges, including placebo effects, variability in training protocols, and concerns regarding replicability, have highlighted the need for rigorous controlled designs, such as sham interventions, to establish the true efficacy of neurofeedback [23,24].

NFB protocols target specific EEG frequency bands to modulate cognitive and behavioral states. Training in lower frequency ranges, such as alpha or alpha/theta protocols, is typically aimed at relaxation and stress reduction, whereas higher frequency training, including the low beta range (12–15 Hz) or sensorimotor rhythm (SMR), is primarily used to modulate attention, focus, and cognitive control [25,26]. SMR training, typically applied over the sensorimotor cortex, aims to increase SMR amplitude and may reduce sensorimotor interference, thereby supporting focused attention and more efficient information processing [27,28]. The SMR rhythm originates from thalamo-cortical networks and is typically enhanced during relaxed wakefulness with reduced motor and sensory excitability [29,30]. NFB-SMR training has been associated with improvements in cognitive performance, including attention, working memory, and memory consolidation, possibly by facilitating cortical integration and inhibitory control within thalamo-cortical circuits [28,31,32]. Numerous studies suggest that SMR neurofeedback may enhance cognitive and behavioral outcomes in both clinical and healthy populations [28,30,32,33,34,35]. However, the extent to which SMR neurofeedback can enhance selective auditory attention and modulate its underlying electrophysiological correlates in healthy adults remains largely unexplored. The aim of this prospective, sham-controlled study was to investigate the effects of repeated SMR neurofeedback training on selective auditory attention in healthy adults aged 25–40 years, using both electrophysiological and behavioral assessments. Specifically, the study examined how SMR training influences the amplitudes and latencies of the N100, N200, and P300 components during an auditory Go/No-Go discrimination task, and performance accuracy on the QuickSIN test, which measures speech-in-noise perception and indirectly engages auditory attention. By integrating electrophysiological and behavioral measures, this study aims to provide a comprehensive understanding of how SMR neurofeedback can modulate neural activity and potentially enhance attentional performance in healthy adults.

## 2. Materials and Methods

### 2.1. Study Design

This prospective study employed a single-blind, sham-controlled factorial design with repeated measures. Participants were randomly assigned to one of two groups: an experimental group receiving sensorimotor rhythm (SMR) neurofeedback training, or a control group undergoing a sham neurofeedback intervention to control for placebo effects. The participants were blinded to group allocation, whereas the experimenter conducting the neurofeedback sessions was aware of group assignments. To prevent analytical bias, the statistician analyzing the data was blinded to group allocation.

### 2.2. Participants

A total of 46 healthy adults (23 women, 23 men) aged 25 to 40 years were recruited from the Institute for Experimental Phonetics and Speech Pathology (Belgrade, Serbia) and the Life Activities Advancement Institute (Belgrade, Serbia). Sample size was determined a priori using G*Power 3.1.9.15. Assuming a medium effect size (Cohen’s f = 0.25), α = 0.05, and desired statistical power of 0.80, the required sample size was calculated as 23 participants per group, totaling 46 participants. Participants were matched for sex and age and allocated to two groups: an experimental group (E), which received the SMR neurofeedback protocol, and a control group (C), which received the sham neurofeedback protocol. Inclusion criteria required that participants report no history of hearing or speech disorders, and no current or past neurological or psychiatric conditions. Exclusion criteria included the use of medications affecting the central nervous system, excessive caffeine or alcohol consumption prior to testing, and any prior experience with neurofeedback training. All participants were right-handed, as assessed by the Edinburgh Handedness Inventory [36], to ensure consistency in lateralization and minimize variability in neural activity related to hemispheric dominance. Prior to the experiment, all participants underwent standard audiological assessment, including pure-tone audiometry (which confirmed that hearing thresholds were within normal limits), speech audiometry, tympanometry, impedance audiometry, and otoacoustic emissions (TEOAE and DPOAE). The study was conducted in the Cognitive Neuroscience Department of the Life Activities Advancement Institute. All participants provided written informed consent prior to enrollment. The study was conducted in accordance with the ethical standards of the Declaration of Helsinki and was approved by the Ethics Committee of the Institute for Experimental Phonetics and Speech Pathology in Belgrade, Serbia (No C-22-01; Date: 10 February 2022).

### 2.3. Procedure

The experiment was structured into three phases: (1) pre-intervention evaluation—the first phase consisted of a pre-intervention evaluation to establish baseline electrophysiological and behavioral measures; (2) intervention phase—the second phase involved the intervention, during which participants underwent either the SMR neurofeedback protocol or a sham neurofeedback protocol; and (3) post-intervention evaluation—the third phase comprised the post-intervention evaluation, including a one-month follow-up, to assess the persistence of any training-related changes. The total duration of the study was 11 to 12 weeks per participant.

Electrophysiological and behavioral assessments, including event-related potentials (ERPs) recording and performance on the QuickSIN (Quick Speech-in-Noise) test, were conducted at five time points: T0 (pre-intervention), T1 (after five sessions), T2 (after ten sessions), T3 (after twenty sessions), and T4 (one-month follow-up, post-intervention). The following sections outline the procedures for ERP recording and the QuickSIN test.

#### 2.3.1. Event-Related Potentials (ERPs) Recording

Cognitive evoked potentials were recorded using a Nihon Kohden Electroencephalograph (model EEG-4314F; Nihon Kohden Corporation, Tokyo, Japan). Silver chloride electrodes were placed on the scalp using collodion as a contact medium. Active electrodes were positioned at frontal (Fz), central (Cz), and parietal (Pz) midline sites according to the international 10–20 system. Reference electrodes were placed on the auricles, and a ground electrode was placed on the forehead. Impedance was maintained below 5 kΩ, with inter-electrode impedance differences not exceeding 1 kΩ. Artifacts were automatically rejected by the software. For each participant, mean amplitudes (μV) and latencies (ms) of the N100, N200, and P300 components were obtained for each electrode site (Fz, Cz, and Pz).

An auditory oddball paradigm was used to elicit ERPs. Pure tones of 1000 Hz (standard, 80%) and 2000 Hz (target, 20%) were presented binaurally at 90 dB SPL via headphones, with irregular inter-stimulus intervals and random order. The stimuli were presented with a duration of 100 ms (including 10 ms rise/fall time). Participants were instructed to ignore the standard tones and to respond as quickly as possible by pressing a button with their dominant hand upon hearing the target tone.

The N100 component is the earliest negative deflection, occurring approximately 90–200 ms after stimulus onset, and was elicited by both target and non-target stimuli, reflecting early sensory processing [37]. In contrast, the N200 component, which was analyzed only for target stimuli, reflects early attentional processes and deviance detection, while the P300 component, also analyzed only for target stimuli, is associated with higher-order cognitive processes related to attention and stimulus evaluation.

To eliminate visual stimulation, participants were seated inside a white, opaque sound-attenuated chamber. All recordings were performed in the morning (9:00–11:00 a.m.) to minimize circadian variability. Participants were instructed to obtain at least eight hours of sleep prior to recording and to refrain from consuming medication, alcohol, or caffeine for at least 24 h before the session.

##### EEG Data Preprocessing and Analysis

Offline preprocessing was performed using EEGLAB (v2021.0) and MATLAB (R2020a). Continuous EEG data (500 Hz sampling rate) were band-pass filtered: 0.5–30 Hz for ERP analysis and 0.5–45 Hz for SMR power analysis. For ERPs, data were epoched from −200 to 800 ms relative to stimulus onset, baseline-corrected (−200 to 0 ms), and automatically artifact-rejected (±75 µV), followed by visual inspection. The number of accepted trials per condition was recorded for each participant, with a minimum of 30 artifact-free trials required for inclusion in the average waveform. Average waveforms were computed for Fz, Cz, and Pz. N100 (90–200 ms), N200 (200–350 ms), and P300 (250–500 ms) were identified as the most negative/positive peaks; amplitudes were measured relative to baseline and latencies as peak times. For SMR power, Cz data were segmented into 2 s epochs (50% overlap), and FFT (Hamming window, 0.5 Hz resolution) was applied. SMR power (12–15 Hz) was averaged per session, expressed as absolute power (µV^2^) and normalized to baseline (first session).

#### 2.3.2. QuickSIN Test

The QuickSIN test is one of the most widely used methods for assessing speech intelligibility in noise, providing a rapid and standardized measure of speech-in-noise perception [38]. The Serbian version of the QuickSIN test was used in the experiment. The test employs predefined test sentences presented in multi-talker “cocktail-party” noise, simulating real-life listening conditions. Sentences are phonetically balanced, grammatically correct, and have a low probability of guessing words due to context. Sentences are presented via loudspeakers or headphones, and participants are instructed to identify and write down all keywords they perceive. Each test list contains six sentences, each with five keywords, yielding a total of 30 keywords per list, and the complete QuickSIN set comprises 21 lists (126 sentences). The signal-to-noise ratio (SNR) decreases progressively across sentences within a list in steps of 5 dB, starting from 25 dB for the first sentence (easiest) to 0 dB for the sixth sentence (most challenging), where speech and noise are at equal intensity. The average SNR at which individuals with normal hearing correctly identify approximately 50% of keywords is +2 dB (i.e., their SNR-50 is 2 dB). The performance is scored based on the number of correctly identified keywords. Because sentences are not repeated and the level of noise varies continuously, the test requires sustained attentional focus. QuickSIN performance reflects not only auditory processing but also the listener’s ability to maintain attention in demanding acoustic conditions, providing a sensitive measure of functional hearing in noise.

In the present study, five sets of four sentences were randomly selected from the 21 lists for each participant, with no sentence repeated across sessions. At each of the five assessment time points, participants listened only to sentences five and six (the two most difficult levels, with the lowest S/N ratios), resulting in 20 keywords per sentence type per session (4 sentences × 5 keywords). Mean values of correctly recognized keywords were recorded for all participants at each session.

#### 2.3.3. Intervention Phase

Following the baseline assessments, participants entered the intervention phase, during which they underwent either the SMR neurofeedback or sham protocol.

##### SMR Neurofeedback Protocol

Neurofeedback training was conducted using BioTrace software (Mind Media BV, Herten, The Netherlands) for the Nexus-10 device (2015 version). The active electrode was placed at Cz, the reference electrode on the right mastoid, and the ground electrode on the left mastoid, according to the international 10–20 system. Cz was chosen as the training site because it is the standard electrode location for SMR (12–15 Hz) neurofeedback, positioned over the sensorimotor cortex where SMR activity is most prominent. In contrast, the auditory oddball paradigm included Fz, Cz, and Pz to capture the full topographical distribution of auditory ERPs, consistent with standard practice in auditory attention research. The goal of the SMR protocol was to train participants to increase SMR amplitude (12–15 Hz). Reward thresholds were individually determined at the start of each session and dynamically adjusted to maintain a success rate between 60% and 80%, which is considered optimal for sustaining motivation and facilitating learning. Before each training session, a 2 min resting EEG recording with eyes open was performed to establish individual baseline SMR levels. Participants were instructed to remain physically relaxed, minimize eye blinks and body movements, and were made aware of common sources of EEG artifacts. Throughout the training, feedback was delivered exclusively via a visual racing game interface, in which the speed of a car was continuously modulated by the participant’s real-time SMR amplitude. No auditory feedback was used.

**Figure 1:**
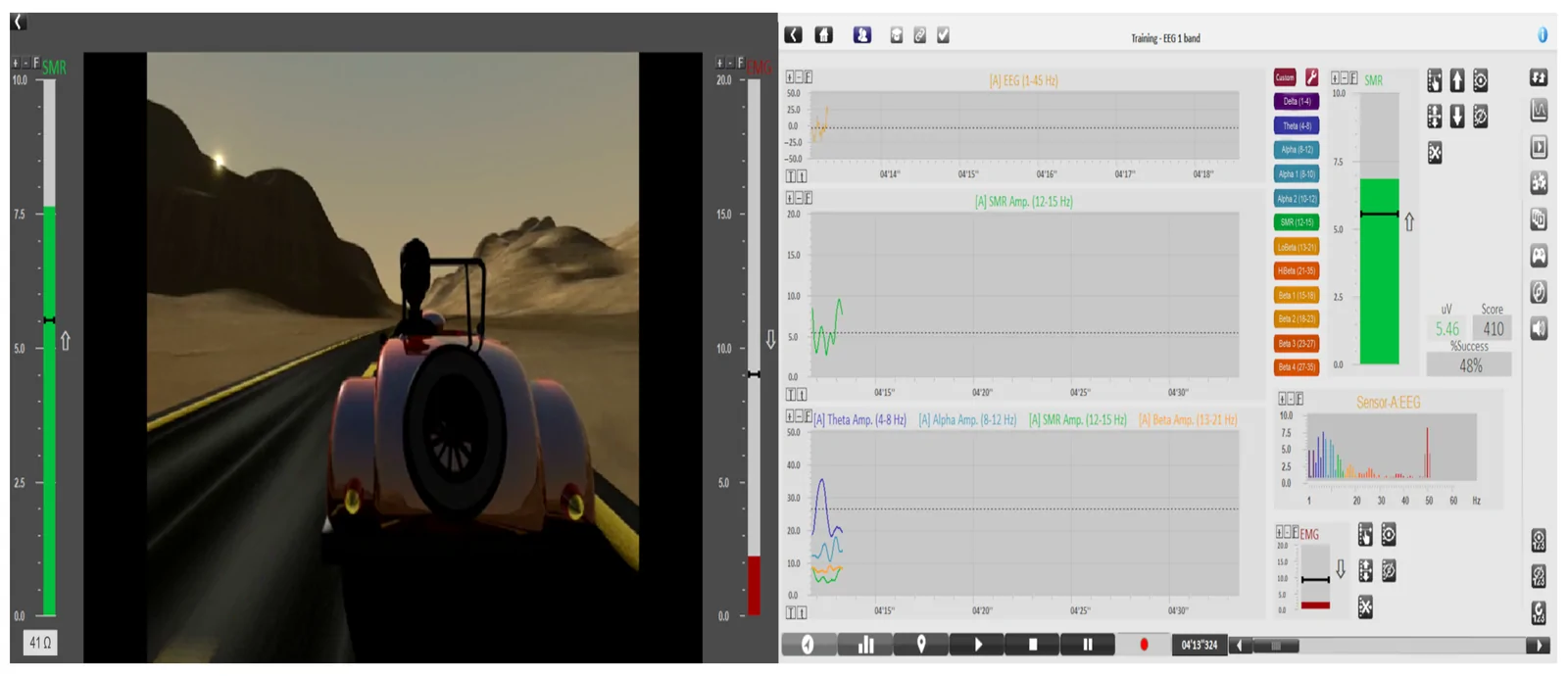
Schematic representation of the visual SMR neurofeedback training interface. The (**left**) panel shows the racing game as presented to the participant, with a car moving along a road. The (**right**) panel illustrates the experimental control software, featuring menu options and a timeline for tracking session parameters. The car’s speed was controlled by SMR amplitude (12–15 Hz) recorded from the Cz electrode, with an adaptive reward threshold adjusted to maintain a 60–80% success rate. No auditory feedback was used.

Visual feedback was presented in real time whenever SMR amplitude exceeded a predefined reward threshold. The video game interface consisted of a simple racing game displayed on a computer monitor. In one of the four games, the participant controlled a car that moved along a two-lane track. The car’s speed was continuously modulated by the SMR amplitude: higher SMR amplitude resulted in faster forward movement, while lower SMR amplitude caused the car to slow down or stop. The goal was to maintain maximum speed, requiring the participant to sustain elevated SMR amplitude throughout each training block. A schematic representation of the game interface is provided in Figure 1.

No auditory signals (e.g., tones or beeps) were used during training; all performance-related feedback was conveyed through the visual movement of the car. This ensured that learning was based solely on visuospatial reinforcement. No explicit cognitive strategy was prescribed, allowing participants to discover, through trial and error, the subjective states associated with optimal SMR modulation. However, based on debriefing after each session, participants commonly reported using strategies such as relaxing their jaw and shoulder muscles, reducing eye blinks, focusing their gaze on a specific point on the screen, or engaging in internal counting. These strategies were recorded informally to monitor consistency across sessions but were not formally analyzed.

When brain activity met the preset criteria, the car moved forward; when activity fell below threshold, the car slowed down or stopped. This provided immediate visual feedback on changes in brain activity. The training was based on operant conditioning principles, whereby desired EEG activity was positively reinforced through successful task performance. Neurofeedback is a learning process during which participants gradually develop the ability to modify targeted EEG patterns based on continuous feedback about their own neural activity.

Reward thresholds were set individually at the beginning of each session and adjusted as necessary during training to maintain a success rate between 60% and 80%. This level of success is considered optimal for maintaining motivation and facilitating learning of self-regulation of EEG activity.

Each participant completed 20 sessions of the EEG protocol (SMR or sham), conducted three times per week. Each session lasted approximately 45 min, including 10 min for preparation (scalp cleaning and electrode placement), 2 min of resting EEG recording at the beginning, 24 min of SMR training divided into four 6 min blocks with 1 min breaks between blocks, and 2 min of resting EEG recording at the end. To minimize circadian variability, all sessions were scheduled at the same time of day for each participant, with an allowable variation of 1 to 2 h.

##### Sham Neurofeedback Protocol

Participants in the control group underwent the same procedure, except that the feedback was not contingent on their actual EEG activity. The sham protocol was designed to control for placebo effects while maintaining the same duration, number of sessions, and experimental environment as the SMR group.

### 2.4. Statistical Analysis

The Statistical Package for the Social Sciences version 24.0 was used for statistical data analysis. Descriptive statistics were computed for all variables and are presented as means with standard deviations (SD) for each group and assessment time point.

To account for the hierarchical structure of the data (repeated measurements nested within participants), we employed Generalized Linear Mixed Models (GLMM) with repeated measures as a fixed factor (Measurement), treating the five time points as a categorical variable. The model included Measurement, Group, and the Measurement × Group interaction as fixed effects, with participants treated as a random intercept to account for between-subject variability. These models were used to analyze the effects of Group (experimental vs. sham control), Session (five time points: pre-intervention, after 5, 10, and 20 sessions, and one-month follow-up), and their interaction on both electrophysiological (ERP latencies and amplitudes) and behavioral (QuickSIN) outcomes. GLMMs properly handle missing data and model within-subject correlations without requiring sphericity assumptions.

To determine whether there was a statistically significant difference between the E and C groups following the NFB treatment, a One-way ANOVA was conducted. As Levene’s test for homogeneity of variance was significant (*p* < 0.05) for all variables, Welch’s test was used for equality of means.

Graphical trends were represented using Excel’s trendline function, with equation parameters and R^2^ values.

## 3. Results

The results are organized into three sections. First, the effects of SMR neurofeedback on the amplitudes and latencies of the N100, N200, and P300 components during the auditory Go/No-Go task are reported. Second, the effects of the training protocol on speech-in-noise perception (which engages auditory selective attention mechanisms), assessed using the QuickSIN test, are presented. Third, results of the One-way ANOVA comparing experimental and control groups at one-month follow-up are reported.

### 3.1. Effects of SMR Neurofeedback on ERP Components

The effects of repeated SMR neurofeedback training on electrophysiological measures were analyzed for the N100, N200, and P300 components during the auditory Go/No-Go task.

To explore the influence of different factors on ERP amplitude and latency for Fz, Cz, and Pz electrodes, we used a Generalized Linear Mixed Model (GLMM) with the following model parameters: type of measurement—repeated measures (Measurement); fixed effects: Measurement and Group and the interaction term Measurement × Group; distribution: Gamma; link function: Log; maximum iterations = 400; degrees of freedom—variable across tests; tests of fixed effects and coefficients—robust estimation. In Table 1, only statistically significant results are presented.

As shown in Table 1, GLMM analysis revealed significant main effects of Measurement for N200 latencies at Cz (F(4, 220) = 3.377, *p* = 0.010) and Pz (F(4, 220) = 4.345, *p* = 0.002), indicating significant changes across sessions regardless of group. The significant Measurement × Group interactions at Cz (F(4, 220) = 3.640, *p* = 0.007) and Pz (F(4, 220) = 3.333, *p* = 0.011) suggest that the experimental and control groups exhibited differential N200 latency changes over the course of training.

For P300 latencies, significant main effects of Measurement were observed at Fz (F(4, 131) = 3.656, *p* = 0.007), Cz (F(4, 149) = 13.143, *p* < 0.001), and Pz (F(4, 135) = 6.907, *p* < 0.001). A significant main effect of Group was found for P300 latencies at Cz (F(1, 20) = 19.443, *p* < 0.001) and Pz (F(1, 20) = 19.443, *p* < 0.001), with the experimental group exhibiting shorter latencies compared to the control group. Significant Measurement × Group interactions were also observed at Fz (F(4, 131) = 2.552, *p* = 0.042), Cz (F(4, 149) = 6.122, *p* < 0.001), and Pz (F(4, 135) = 6.825, *p* < 0.001), confirming that the experimental group showed progressive latency reductions across sessions, whereas the control group did not.

No significant main effects or interactions were found for ERP amplitudes at any electrode site (all *p* > 0.05).

**Figure 2:**
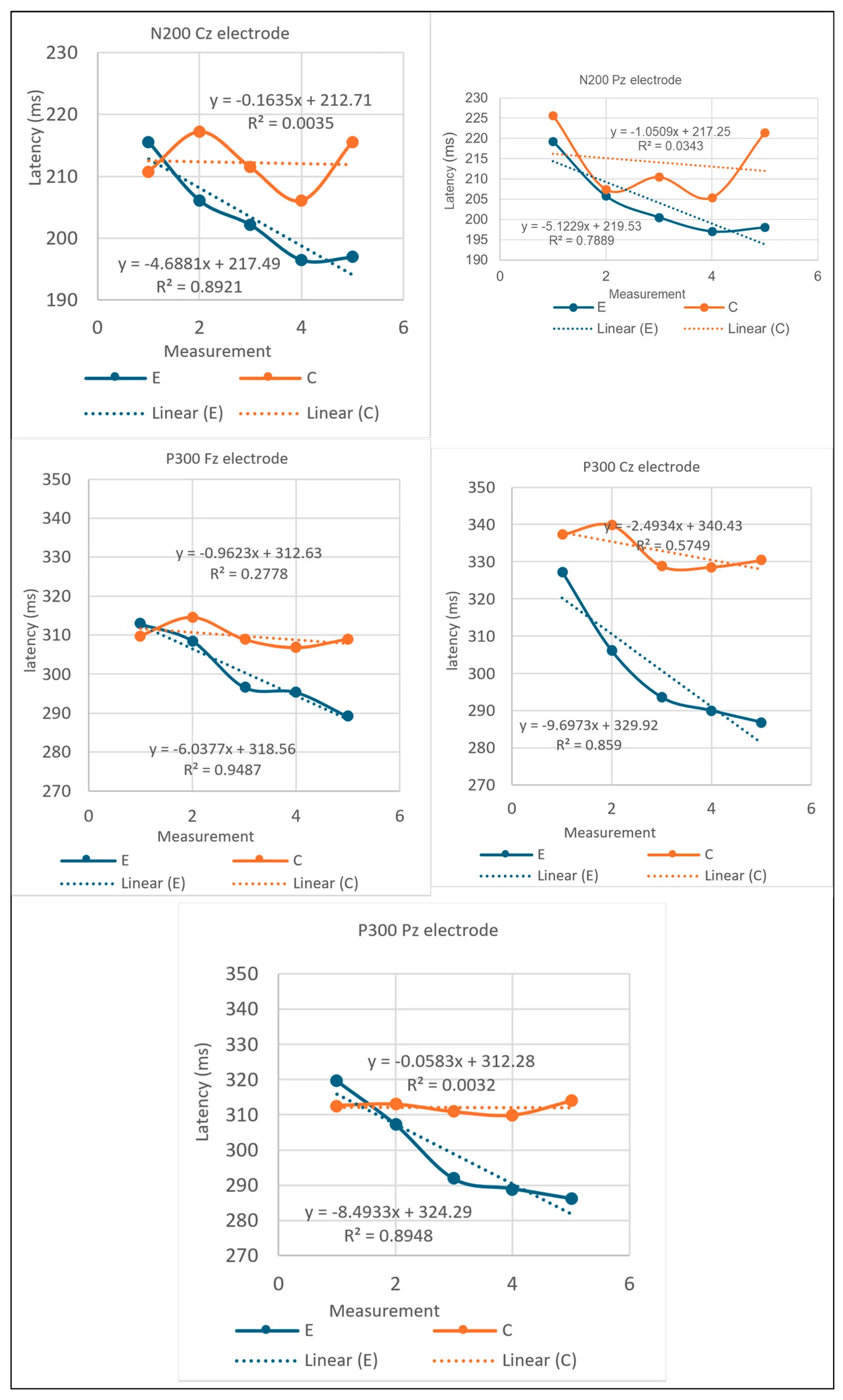
Changes in N200 and P300 latencies across five assessment time points for the experimental (E) and control (C) groups. Data are shown for: N200 at Cz, N200 at Pz, P300 at Fz, P300 at Cz, and P300 at Pz. Solid lines represent observed values; dashed lines represent linear trends. R^2^ values and regression equations are presented for each electrode site.

For the statistically significant latency effects, graphical data and trend lines are presented in Figure 2.

Figure 2 shows the changes in N200 and P300 latencies across five assessment time points (pre-intervention, after 5, 10, and 20 sessions, and one-month follow-up) for both the experimental (E) and control (C) groups.

For N200 latency, the experimental group exhibited a progressive linear decrease at both Cz (y = −4.6881x + 217.4, R^2^ = 0.8921) and Pz (R^2^ = 0.8094), while the control group showed relatively stable trends (Cz: R^2^ = 0.1635; Pz: R^2^ = 0.0343).

For P300 latency, the experimental group showed consistent linear reductions at Fz (y = −0.9623x + 312.63, R^2^ = 0.2778), Cz (y = −2.4934x + 340.43, R^2^ = 0.5749), and Pz (y = −8.4933x + 324.29, R^2^ = 0.8948). In contrast, the control group exhibited stable latencies at Cz and Pz, with a slight increase at Fz (y = 6.0377x + 318.56, R^2^ = 0.9487).

Overall, the negative trend lines for the experimental group indicate dose-dependent latency shortening with repeated SMR neurofeedback training.

### 3.2. The Effects of the SMR Training Protocol on Auditory Selective Attention

The effects of SMR neurofeedback training on speech-in-noise perception, which engages auditory selective attention mechanisms, were assessed using the QuickSIN test.

For the QuickSIN results, a Generalized Linear Mixed Model (GLMM) was used with the following model parameters: type of measurement—repeated measures (Measurement); fixed effects: Measurement and Group and the interaction term Measurement × Group; distribution: Normal; link function: Identity; maximum iterations = 400; degrees of freedom—variable across tests; tests of fixed effects and coefficients—robust estimation. The obtained results are presented in Table 2.

As shown in Table 2, GLMM analysis revealed significant main effects of Measurement for both Sentence 5 (F(4, 131) = 46.461, *p* < 0.001) and Sentence 6 (F(4, 220) = 198.359, *p* < 0.001), indicating significant improvements in speech perception across sessions regardless of group.

A significant main effect of Group was found for both Sentence 5 (F(1, 9) = 5.243, *p* = 0.049) and Sentence 6 (F(1, 11) = 8.492, *p* = 0.015), with the experimental group outperforming the control group.

Most importantly, significant Measurement × Group interactions were observed for both Sentence 5 (F(4, 131) = 22.029, *p* < 0.001) and Sentence 6 (F(4, 220) = 22.029, *p* < 0.001), indicating that the experimental group showed greater improvement over time compared to the control group.

**Figure 3:**
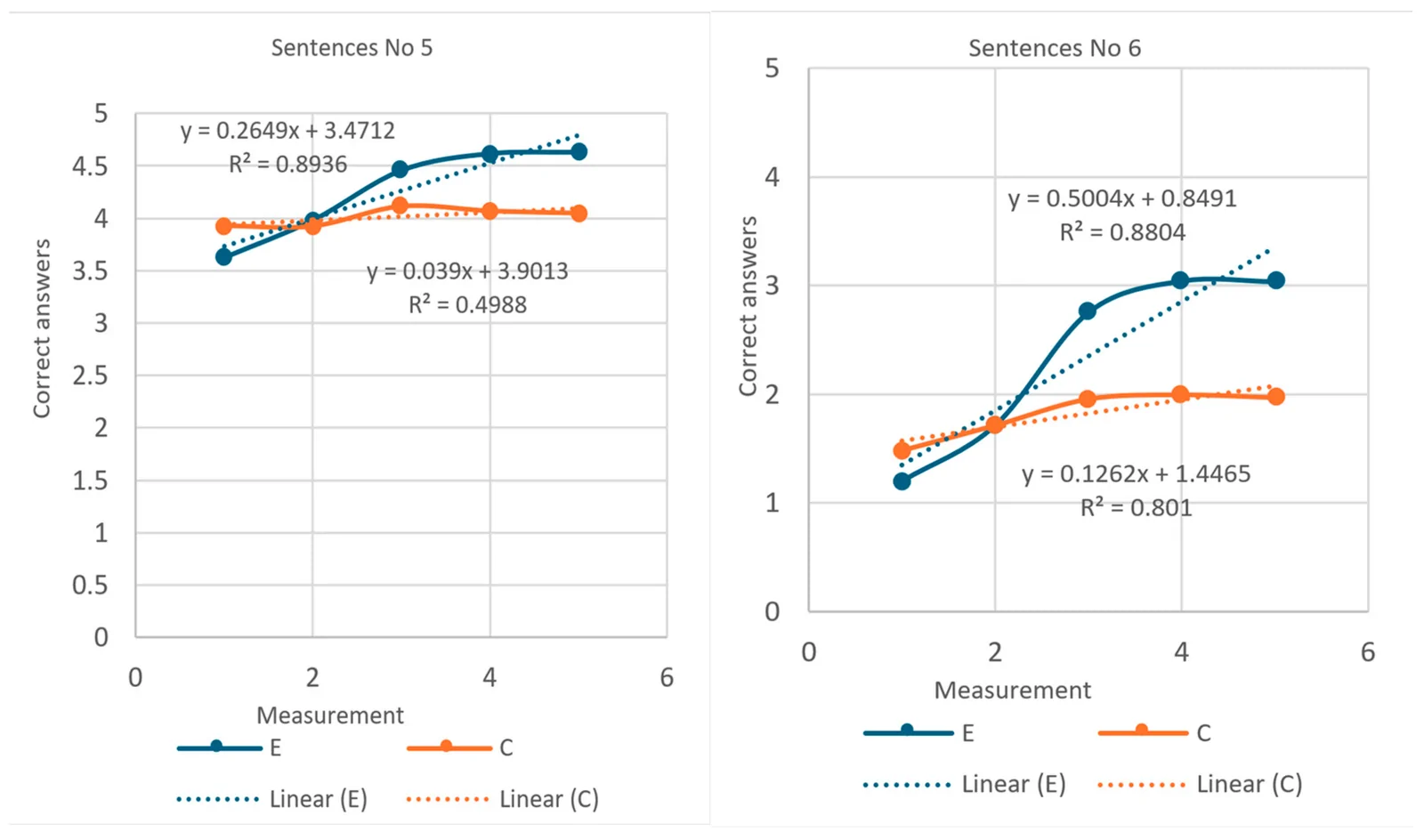
QuickSIN performance for Sentences 5 and 6.

For the QuickSIN performance, graphical data and trend lines for Sentences 5 and 6 are presented in Figure 3.

As shown in Figure 3, for Sentence 5, the experimental group exhibited a marked linear increase in the number of correctly recognized keywords across sessions (y = 0.2649x + 3.4712, R^2^ = 0.9999). In contrast, the control group showed only a minimal increase over time (y = 0.039x + 3.9013, R^2^ = 0.4988).

For Sentence 6, the experimental group also showed a pronounced linear improvement (y = 1.262x + 1.4465, R^2^ = 0.801), while the control group exhibited a modest increase (y = 0.5004x + 0.8491, R^2^ = 0.8804). The steeper slope for the experimental group indicates greater training-induced improvement in speech perception in noise, particularly for the more difficult sentence type (Sentence 6).

### 3.3. One-Month Follow-Up Group Comparisons

To examine whether there was a statistically significant difference between the experimental (E) and control (C) groups after the completion of SMR neurofeedback training, we conducted a one-way ANOVA. Since Levene’s test for homogeneity of variance was significant (*p* < 0.05) for all variables, we used the Welch test for equality of means. The results are presented in Table 3.

One-way ANOVA revealed significant group differences at the one-month follow-up for P300 latencies at Cz and Pz, as well as for QuickSIN performance on both Sentence 5 and Sentence 6 (all *p* < 0.01). No significant differences were observed for N200 latencies at Cz or Pz, or for P300 latencies at Fz (all *p* > 0.05).

## 4. Discussion

The present study investigated the effects of repeated sensorimotor rhythm (SMR) neurofeedback training on auditory selective attention in healthy adults aged 25–40 years.

Electrophysiological results revealed that SMR training statistically significantly reduced latencies of the N200 and P300 components in a dose-dependent manner, particularly over the Cz and Pz regions. No significant amplitude effects were observed for any ERP component. Behavioral results demonstrated a linear improvement in speech perception in noise (QuickSIN test), a task that indirectly engages auditory selective attention mechanisms, as a function of training sessions, with significant group differences favoring the experimental group. These effects were maintained at the one-month follow-up. These findings indicate that SMR neurofeedback enhances auditory selective attention through both electrophysiological and behavioral mechanisms in a dose-dependent manner.

Beyond SMR (12–15 Hz) training over the sensorimotor cortex, neurofeedback studies have targeted other frequency bands and brain regions. For instance, Bagherzadeh et al. [39] used MEG neurofeedback to train subjects to manipulate alpha power asymmetry over left versus right parietal cortex, demonstrating a causal role of alpha synchrony in modulating spatial attention and visual processing. Notably, the training led to persistent attentional biases and corresponding changes in sensory evoked responses, suggesting that NFB effects can propagate through large-scale cortical networks. Similarly, Shim et al. [40] reported that EEG neurofeedback training targeting auditory selective attention improved speech-in-noise performance, with time-frequency analysis revealing increased beta oscillation (reflecting top-down anticipatory processing) and enhanced alpha modulation (indicating improved spatial inhibitory processing) in fronto-parietal and temporal networks. Together, these studies support the view that neurofeedback can induce neuroplastic changes extending beyond the trained frequency band and cortical site, involving distributed brain networks critical for attention and sensory processing.

### 4.1. Effects of SMR Training on ERP Components

In this study, SMR training induced a linear, dose-dependent reduction in N200 latencies at the Cz and Pz electrodes, and in P300 latencies at the Fz, Cz, and Pz electrodes. These findings indicate accelerated stimulus discrimination (N200) and faster cognitive processing (P300) following SMR training. The N200 component is considered a marker of stimulus discrimination and attentional allocation [41]; thus, its increased amplitude in the experimental group suggests improved attentional engagement after training.

The P300 component is a well-established neurophysiological marker of attentional resource allocation and stimulus evaluation speed [14]. In the present study, SMR training resulted in a linear, dose-dependent reduction in P300 latencies across all three electrode sites, indicating faster cognitive processing. This finding is consistent with previous reports that shorter P300 latencies are associated with more efficient stimulus classification [42,43].

No significant amplitude effects were observed for any ERP component. The absence of significant amplitude changes may reflect the relatively short training duration (20 sessions) or the healthy high-functioning sample, which may have limited ceiling effects. Alternatively, amplitude measures may be more subject-dependent and characterized by greater inter-individual variability than latency measures, making it more difficult to detect statistically significant changes [4].

The absence of significant N100 effects in the present study is noteworthy. While some studies have reported N100 latency reductions following SMR training [28], our findings suggest that SMR training may exert its primary effects on later, more cognitive stages of auditory processing (N200, P300) rather than on early sensory processing (N100). This is consistent with the notion that SMR training modulates sensorimotor and attentional networks rather than primary sensory processing [25,28].

### 4.2. Effects of SMR Training on Behavioral Performance

Performance on the QuickSIN test, a measure of speech perception in noise that indirectly engages auditory selective attention mechanisms [9], showed a linear improvement in the experimental group across training sessions. Statistical analysis revealed significant main effects of Measurement and Group, along with a significant Measurement × Group interaction, for both sentence types. The significant group effect and the observed linear improvement support the notion that SMR training enhances the ability to filter relevant auditory information in complex acoustic environments [28].

The trend toward improvement in the control group, although not statistically significant, likely reflects non-specific effects such as test–retest learning or attention to the task environment [8]. This underscores the importance of sham-controlled designs in neurofeedback research [23,24]. The observed behavioral improvements are consistent with studies showing that SMR training enhances attention and working memory in healthy adults [28,30,33]. Moreover, the dose-dependent nature of the effect, with progressive improvement from 5 to 20 sessions, supports the notion that neurofeedback is a skill-learning process requiring repeated practice [44].

Our QuickSIN findings are also consistent with previous neurofeedback studies that specifically examined speech-in-noise outcomes with electrophysiological evidence. Kim et al. [45] demonstrated that EEG-based neurofeedback training of auditory selective attention enhanced cortical responses to target speech and improved performance on a post-training speech-in-noise task. Similarly, Shim et al. [40] reported that EEG neurofeedback training targeting auditory selective attention improved speech-in-noise performance, with time-frequency analysis revealing increased beta oscillation, reflecting top-down anticipatory processing.

Our results are also consistent with Shim et al. [40], who demonstrated that EEG neurofeedback targeting auditory selective attention improved speech-in-noise performance, with concomitant changes in beta and alpha oscillations. Similarly to our findings, their study showed that NFB effects extend beyond the trained frequency band, involving fronto-parietal networks critical for auditory attention. Together with our results, these studies support the view that neurofeedback, particularly SMR training, can induce neuroplastic changes in auditory attention networks, leading to better speech perception in noisy environments.

### 4.3. Dose-Dependent Effects and Stability of Outcomes

A notable finding of this study is the dose-dependent nature of the observed effects. For both electrophysiological (N200, P300 latencies) and behavioral (QuickSIN) measures, improvements progressed as a function of the number of training sessions. This pattern supports the view that neurofeedback effects are cumulative and reflect learning-related neuroplastic changes [24].

Importantly, improvements were maintained at the one-month follow-up (t4) for behavioral measures and for most electrophysiological measures, suggesting relative stability of training outcomes. This is in line with studies demonstrating long-term maintenance of NFB effects [46,47,48].

The robustness of these findings is supported by the methodological quality of the present study. The use of a single-blind, sham-controlled factorial design with repeated measures strengthens the validity of the findings by controlling for placebo effects and non-specific factors [23,24]. The prospective design ensured that all assessments and interventions were conducted according to a predefined protocol, minimizing the risk of bias. Additionally, the inclusion of multiple assessment time points (pre-intervention, after 5, 10, and 20 sessions, and at one-month follow-up) allowed for the examination of dose-dependent effects, a key contribution of this study.

## 5. Conclusions

This study demonstrates that repeated sessions of SMR neurofeedback training enhance auditory selective attention in healthy adults aged 25–40 years. The training resulted in dose-dependent reductions in N200 and P300 latencies, reflecting faster neural processing of auditory stimuli. No significant amplitude effects were observed for any ERP component. Behavioral results demonstrated a linear improvement in speech perception in noise (QuickSIN test), a task that indirectly engages auditory selective attention mechanisms, as a function of training sessions, with significant group differences favoring the experimental group. These findings highlight the potential of SMR neurofeedback as a non-invasive, dose-dependent intervention for improving attentional performance and auditory processing in healthy populations, with effects persisting at least one month post-training.

### Limitations and Future Directions

Several limitations should be acknowledged. The sample consisted exclusively of healthy, right-handed, normal-hearing adults from Serbia, which limits the generalizability of our findings to other populations, including individuals with hearing impairments, left-handed individuals, or those from different cultural and linguistic backgrounds. Future studies should replicate these findings in more diverse samples to establish the broader applicability of SMR neurofeedback training for enhancing auditory attention. Additionally, the sample size may have limited statistical power to detect smaller effects, particularly for amplitude measures where no significant changes were observed, which may reflect the relatively small sample size or the high inter-individual variability typically observed in ERP amplitude measures. Furthermore, inclusion criteria relied on self-report for neurological and psychiatric conditions, which may have introduced selection bias. Moreover, while our study included a sham control group, the absence of an active control group precludes conclusions about the frequency-specific effects of SMR training as opposed to neurofeedback in general. The one-month follow-up also does not address the long-term durability of training effects. Finally, the dose-dependent effects observed here suggest that the number of sessions critically influences outcomes, and future research should explore optimal training schedules, examine whether SMR training generalizes to other cognitive domains, and extend this protocol to populations with attentional deficits to elucidate its clinical applicability.

## Figures and Tables

**Table 1:** Generalized Linear Mixed Model (GLMM) results for N200 and P300 latencies showing statistically significant main effects and interactions.

Source	F	df1	df2	Significance
N200 latency for Cz electrode				
Corrected model	3.762	9	161	0.000
Measurement	3.377	4	220	0.010
Measurement × Group	3.640	4	220	0.007
N200 latency for Pz electrode				
Corrected model	4.401	9	149	0.000
Measurement	4.345	4	220	0.002
Measurement × Group	3.333	4	220	0.011
P300 latency for Fz electrode				
Corrected model	3.609	9	113	0.001
Measurement	3.656	4	131	0.007
Measurement × Group	2.552	4	131	0.042
P300 latency for Cz electrode				
Corrected model	11.409	9	106	0.000
Measurement	13.143	4	149	0.000
Group	19.443	1	20	0.000
Measurement × Group	6.122	4	149	0.000
P300 latency for Pz electrode				
Corrected model	11.409	9	106	0.000
Measurement	13.143	4	149	0.000
Group	19.443	1	20	0.000
Measurement × Group	6.122	4	149	0.000
P300 latency for Fz electrode				
Corrected model	6.099	9	103	0.000
Measurement	6.907	4	135	0.000
Measurement × Group	6.825	4	135	0.000

*Note*. The ‘×’ symbol denotes an interaction between factors.

**Table 2:** Generalized Linear Mixed Model (GLMM) results for QuickSIN speech-in-noise performance (Sentences 5 and 6) showing statistically significant main effects and interactions.

Source	F	df1	df2	Significance
Sentences No 5				
Corrected model	43.221	9	57	0.000
Measurement	46.461	4	131	0.000
Group	5.243	1	9	0.049
Measurement × Group	22.029	4	131	0.000
Sentences No 6				
Corrected model	131.564	9	90	0.000
Measurement	198.359	4	220	0.000
Group	8.492	1	11	0.015
Measurement × Group	22.029	4	220	0.000

*Note*. The ‘×’ symbol denotes an interaction between factors.

**Table 3:** One-way ANOVA (Welch test) results comparing experimental and control groups at one-month follow-up.

		Statistic	df1	df2	Sig.
N200 Cz	Welch	2.888	1	38.889	0.097
N200 Pz	Welch	3.330	1	38.186	0.076
P300 Fz	Welch	3.817	1	34.437	0.059
P300 Cz	Welch	17.603	1	33.238	0.000
P300 Pz	Welch	8.224	1	33.107	0.007
Sentences 5	Welch	36.766	1	26.465	0.000
Sentences 6	Welch	30.657	1	40.220	0.000

## Data Availability

The data presented in this study are available on request from the corresponding author (the data are not publicly available due to privacy or ethical restrictions).

## Abbreviations

The following abbreviations are used in this manuscript:

NFB	Neurofeedback
SMR	Sensorimotor rhythm
ERP	Event-related potential
ERPs	Event-related potentials
EEG	Electroencephalography
TMS	Transcranial magnetic stimulation
TES	Transcranial electrical stimulation
SIN	The speech-in-noise
QuickSIN	Quick speech-in-noise
SNR	Signal-to-noise ratio
GLMM	General linear mixed model
